# Chronic administration of prebiotics and probiotics ameliorates pathophysiological hallmarks of Alzheimer’s disease in a APP/PS1 transgenic mouse model

**DOI:** 10.3389/fphar.2024.1451114

**Published:** 2024-08-06

**Authors:** Daniele Lana, Chiara Traini, Irene Bulli, Giorgia Sarti, Giada Magni, Selene Attorre, Maria Grazia Giovannini, Maria Giuliana Vannucchi

**Affiliations:** ^1^ Section of Clinical Pharmacology and Oncology, Department of Health Sciences, University of Florence, Florence, Italy; ^2^ Research Unit of Histology and Embryology, Department of Experimental and Clinical Medicine, University of Florence, Florence, Italy; ^3^ Cnr — Istituto di Fisica Applicata “Nello Carrara”, Sesto Fiorentino, Italy; ^4^ Section of Anatomic Pathology, Department of Health Sciences, University of Florence, Florence, Italy

**Keywords:** astrocytes, ball-and-chain microglia, neurodegeneration, beta-amyloid, CA1 hippocampus, CA3 hippocampus, phagocytosis, microbiota

## Abstract

**Introduction:** The gut microbiota (MB), although one of the main producers of Aβ in the body, in physiological conditions contributes to the maintainance of a healthy brain. Dysbiosis, the dysbalance between Gram-negative and Gram-positive bacteria in the MB increases Aβ production, contributing to the accumulation of Aβ plaques in the brain, the main histopathological hallmark of Alzheimer’s disease (AD). Administration of prebiotics and probiotics, maintaining or recovering gut-MB composition, could represent a nutraceutical strategy to prevent or reduce AD sympthomathology. Aim of this research was to evaluate whether treatment with pre- and probiotics could modify the histopathological signs of neurodegeneration in hippocampal CA1 and CA3 areas of a transgenic mouse model of AD (APP/PS1 mice). The hippocampus is one of the brain regions involved in AD.

**Methods:** Tg mice and Wt littermates (Wt-T and Tg-T) were fed daily for 6 months from 2 months of age with a diet supplemented with prebiotics (a multi-extract of fibers and plant complexes, containing inulin/fruit-oligosaccharides) and probiotics (a 50%–50% mixture of *Lactobacillus rhamnosus* and *Lactobacillus paracasei*). Controls were Wt and Tg mice fed with a standard diet. Brain sections were immunostained for Aβ plaques, neurons, astrocytes, microglia, and inflammatory proteins that were evaluated qualitatively and quantitatively by immunofluorescence, confocal microscopy and digital imaging with ImageJ software.

**Results:** Quantitative analyses demonstrated that: 1) The treatment with pre- and probiotics significantly decreased Aβ plaques in CA3, while in CA1 the reduction was not significant; 2) Neuronal damage in CA1 Stratum Pyramidalis was significantly prevented in Tg-T mice; no damage was found in CA3; 3) In both CA1 and CA3 the treatment significantly increased astrocytes density, and GFAP and IBA1 expression, especially around plaques; 4) Microglia reacted differently in CA1 and CA3: in CA3 of Tg-T mice there was a significant increase of CD68+ phagocytic microglia (ball-and-chain phenomic) and of CX3CR1 compared with CA1.

**Discussion:** The higher microglia reactivity could be responsible for their more efficient scavenging activity towards Aβ plaques in CA3 in comparison to CA1. Treatment with pre- and probiotics, modifying many of the physiopathological hallmarks of AD, could be considered an effective nutraceutical strategy against AD symptomatology.

## 1 Introduction

The statement by the Greek physician Hippocrates more than 2,000 years ago “all diseases begin in the gut” is still intriguing and continues to influence medical doctors and researchers (Cryan et al., 2019). However, the questions whether and how the gut and its microbiota contribute to the onset and/or progression of neurodegeneration remain unanswered. What we do know is that neurological disorders such as AD, Parkinson’s disease (PD), and amyotrophic lateral sclerosis are characterized by the gradual accumulation of abnormal proteins in the central nervous system (Jucker and Walker, 2018), and one of the histopathological hallmarks of AD is the accumulation of misfolded Aβ peptides that organize into fibrils that precipitate and form plaques in brain parenchyma (Glenner and Wong, 1984; Koyama et al., 2012). Several studies have shown that the MB-gut system is a major site of Aβ production (Pallebage-Gamarallage et al., 2012), and, together with the diet regulates the presence of Aβ in the intestinal epithelium (Galloway et al., 2019). The accumulation of intestinal APP from the early stages of AD (Brandscheid et al., 2017) is mainly characterized by the increase of Gram-negative and decrease of Gram-positive bacteria (Kowalski and Mulak, 2019). Dysbiosis, the quantitative and qualitative changes of MB, causes morphological modifications and dysfunction of both the intestinal barrier, involved in neuroinflammatory responses (Iadecola et al., 1999), and of the blood brain barrier (BBB) (Mayer and Tillisch, 2011; Sharon et al., 2016; Brown, 2019). How dysbiosis contributes to neuronal degeneration remains to be determined, but accumulating evidences show that APP/PS1 transgenic mice produce excessive amounts of Aβ, harbor an altered microbiota (Harach et al., 2017) and show neurodegeneration (McGowan et al., 1999). Studies on the composition of the gut microbiota in APP/PS1 mice demonstrate that levels of Aβ in the CNS increase and spatial learning and memory is impaired (Shen et al., 2017). Furthermore, patients with neurodegeneration have dysbiosis (Kesika et al., 2021).

Aberrant accumulation of Aβ in the gut and activation of intestinal innate immunity appear before the onset of CNS neuroinflammation in AD mice, an amyloid-β protein precursor (AβPP) overexpressing transgenic mouse model (Semar et al., 2013). Similarly, intestinal dysbiosis, intestinal epithelial barrier dysfunction, and vascular Aβ deposition in the intestine occur before the onset of cerebral Aβ deposition in AD Tg2576 mice. Aβ deposition is also detected in intestinal autopsies of AD patients (Honarpisheh et al., 2020), suggesting that the accumulation of Aβ in the gut precedes that in the brain. When Aβ reaches the brain, its overload or defective clearance may cause its accumulation, promoting fibril organization and plaque deposition (Brown, 2019).

These data suggest that specific individualized nutritional interventions could be an effective strategy to modify Aβ production and aggregation. However, there is a lack of research aimed at clarifying the relationship between gut dysbiosis, gut Aβ accumulation, and AD onset. Early manipulation of gut physiology and microbiota to possibly prevent AD pathology needs further investigation.

Neuronal loss and memory impairment in AD are mainly associated with accumulation of extracellular Aβ and intracellular deposits of hyperphosphorylated tau. The presence of microglia around plaques and their interaction with Aβ has been extensively documented both in AD patients and in transgenic mouse models of AD (Stalder et al., 1999; Serrano-Pozo et al., 2014), but their exact role is not yet understood. Microglia, rapidly recruited around newly formed plaques (Meyer-Luehmann et al., 2008) express receptors capable of binding and phagocytosing Aβ (Lee and Landreth, 2010; Doens and Fernández, 2014). However, in later stages of the disease microglia lose their ability to remove and destroy Aβ (Krabbe et al., 2013). Thus, microglia activation seems to play a dual role in the pathogenesis of AD: first, reducing Aβ accumulation by increasing its phagocytosis, but later contributing to neurotoxicity and synapse loss by triggering various proinflammatory cascades (Sarlus and Heneka, 2017).

Under physiological conditions, astrocytes exhibit their classical morphology, protruding their processes to establish millions of contacts with synapses and vascular capillaries to modulate vital functions, maintain homeostasis, and provide trophic support to neurons (Schiweck et al., 2018). Astrocytes become reactive when challenged by various insults, such as BBB disruption in AD (Erickson and Banks, 2013). Reactive astrocytes are important components of the plaque microenvironment in the brains of AD patients, infiltrating and enveloping Aβ deposits with their processes (Perez-Nievas and Serrano-Pozo, 2018). Reactive astrocytes around Aβ plaques exhibit high phagocytic activity and activated autophagy (Yue and Hoi, 2023), while expressing and releasing cytokines that alter the permeability and organization of the BBB (Abbott, 2002), one of the early events in the pathogenesis of AD. Alterations of the BBB, as those caused by dysbiosis, allow the passage of proinflammatory factors, of immune cells from the periphery, of peptides such as Aβ, changing the composition of brain environment and brain cell homeostasis. These data suggest that dysbiosis-dependent changes of astrocytic functions may be responsible for decreased disposal of Aβ peptides, increasing Aβ plaque formation (Wang et al., 2017). However, the idea that astrocytes activation is always a negative phenomenon is rapidly changing, giving way to the new concept of a more complex and more diverse role of astrocytes in various neuropathological disorders (Verkhratsky et al., 2013; Burda and Sofroniew, 2014). Reactive astrocytes show functional diversity in AD pathology, promoting neuroprotection through Aβ degradation and clearance but also contributing to Aβ-triggered pathological processes. The gut microbiota can modulate and suppress the inflammatory state of astrocytes, with important consequences for neuroinflammation (Rothhammer et al., 2018). An in-depth characterization of the mechanisms that control astrocytes and the role of prebiotics and probiotics on their phenotypic modification is still lacking.

The present work focused on the study of the effects of a diet enriched in prebiotics and probiotics on the alterations of neurons and glia in the Stratum Pyramidale (SP) and Stratum Radiatum (SR) of hippocampal CA1 and CA3 of APP/PS1 mice, a transgenic model of AD. The pre- and probiotics were administered together since prebiotics are substrates of many bacteria strains such as the Firmicutes, that decrease with age (Cattaneo et al., 2017; Galloway et al., 2019; Sun et al., 2020), and sustain the survival of the Lactobacilli present in our probiotic preparation. The results of this research will help to understand the beneficial effects of pre- and probiotics on the hallmarks of AD pathogenesis. As the diet is one of the most effective approaches to modify the microbiota, food-based therapies, influencing its composition, can modify the function of the central nervous system with very few, if any, side effects.

## 2 Materials and methods

### 2.1 Animals

Male and female APP/PS1 mice (8 months old) were bred and treated in the animal house facility of the University of Florence by the team of Prof. Vannucchi’s. The experimental protocols were approved by the Italian Ministry of Health (Aut. N. 53/2022-PR). The authors further attest that all efforts were made to minimize the number of animals used and their suffering, as reported in the Guidelines McGill Module-1. APP/PS1 mice have human transgenes for APP, bearing the Swedish mutation, and PSEN1, containing an L166P mutation (Radde et al., 2006; Maia et al., 2013). The main feature of this model is the very rapid development of amyloid deposition in the brain. Mice display amyloid plaques in the cortex and hippocampus already at 3 months of age. Animals were housed at 23 ± 1°C under a 12 h light-dark cycle (lights on at 07:00) and were fed a standard laboratory diet with ad libitum access to water.

The following groups were used in the study:

- Wild type mice (Wt): littermate mice that do not possess gene mutations.
- Wt mice + pre- and probiotics (Wt-T): littermate mice, administered with the pre- and probiotic enriched diet.
- Transgenic mice (Tg): APP/PS1 mice that have human transgenes for APP and PSEN1.
- Tg mice + pre- and probiotics (Tg-T): APP/PS1 mice, administered with the pre- and probiotic enriched diet.

Wt-T and Tg-T mice were fed with a pre- and probiotics enriched diet for 6 months, from the end of the 2 month to the end of the 8 month of age. Pre- and probiotics were administered orally via food and water and were made available to the mice for a total of 12 h per day. The probiotic was provided by Synbiotec srl (Camerino, MC) and was a mixture of L. rhamnosus IMC 501 and L. paracasei IMC 502 (50:50, bacterial density 109 cells/g). Prebiotics: a multi-extract of fibers and plant complexes, containing inulin/FOS. Probiotics: a 50%–50% mixture of the bacteria strains: *Lactobacillus* rhamnosus and *Lactobacillus* paracasei. The weight and food intake of the mice was monitored daily.

### 2.2 Samples preparation

Mice of the appropriate age (8 months) were anesthetized (ketamine-dexmedetomidine solution, 80–120 mg/Kg + 0.5–1.0 mg/Kg, respectively, s.c.), and euthanized by guillotine. Subsequently, the brain was extracted and divided sagittally in two. One of the half brains was fixed overnight with ice-cold paraformaldehyde (4% paraformaldehyde in phosphate-buffered saline, PBS, pH 7.4). After 48 h of cryoprotection in 18% sucrose/PBS, the emibrain was immersed in a solution of isopentane at −50°C for 40 s and then cut in 40 μm-thick brain coronal sections with a cryostat. Sections were stored at −20°C in anti-freezing solution (40% PBS, 30% Ethylene-Glycol, 30%, Glycerol, v/v) until immunohistochemistry. The other half brain was quickly frozen and kept at −80°C for further analyses.

### 2.3 Immunohistochemistry

Immunohistochemistry was performed on mice brain coronal sections at the dorsal hippocampus level (−1,700 µm, posterior from Bregma) with the free-floating method (Giovannini et al., 2002; Lana et al., 2014). The antibodies used (see Table 1) and the protocols of the different immunostainings are reported below.

**TABLE 1:** Antibodies used for immunohistochemistry. All antibodies are diluted in BB solution.

Antibodies used for immunohistochemistry							
Target	Antigen	Supplier	Catalog #	Antibody	Host	Usage	Diluition
Beta-amyloid plaques	Abeta	Biolegend (Dedham, MA, United States)	39320-200	Monoclonal	Ms	Primary	1:200 5 μg/mL
Neurons	NeuN	Millipore (Billerica, MA, United States)	MAB377	Monoclonal	Ms	Primary	1:400 2.5 μg/mL
Astrocytes	GFAP	Millipore	MAB3402X	Monoclonal	Ms	Primary Alexa Fluor 488 conjugated	1:500 2 μg/mL
Total microglia	IBA1	Wako (Osaka, JP)	016-20001	Policlonal	Rb	Primary	1:300 1.67 μg/mL
Total microglia	IBA1	Wako	011-27991	Policlonal	Gt	Primary	1:200 3 μg/mL
CD68	CD68	AbCam (Cambridge, United Kingdom)	ab125212	Monoclonal	Rb	Primary	1:200 2.5 μg/mL
CX3CR1	CX3CR1	AbCam	ab308613	Monoclonal	Rb	Primary	1:200 2.4 μg/mL
Rabbit FC	Rabbit FC	Thermo Fisher (Waltham, MA, United States)	A21206	Polyclonal	Dn	Secondary Alexa Fluor 488	1:400 5 μg/mL
Mouse FC	Mouse FC	Thermo Fisher	A31570	Polyclonal	Dn	Secondary Alexa Fluor 555	1:400 5 μg/mL
Rabbit FC	Rabbit FC	Thermo Fisher	A31577	Polyclonal	Gt	Secondary Alexa Fluor 635	1:400 5 μg/mL
Goat FC	Goat FC	Thermo Fisher	A21082	Polyclonal	Dn	Secondary Alexa Fluor 635	1:400 5 μg/mL

Before incubation with primary antibodies, all sections were incubated for 60 min with Blocking Buffer (BB) containing 10% Normal Goat Serum (Product Code: S-1000, Vector, Burlingame, CA, United States) in PBS-TX (0.3% Triton X-100 in PBS). All antibodies were dissolved in BB.

#### 2.3.1 NeuN + GFAP + IBA1 triple labelling immunohistochemistry

*Day 1*: After BB, sections were incubated overnight at 4°C in a solution with two primary antibodies, a mouse anti-NeuN antibody to immunostain neurons (1:400 in BB) and a rabbit anti-IBA1 antibody to immunostain total microglia (1:300 in BB).

*Day 2*: Sections were incubated for 2 h at room temperature in the dark with AlexaFluor 635 goat anti-rabbit secondary antibody (1:400 in BB) and then for 2 h with a solution of AlexaFluor 635 goat anti-rabbit secondary antibody (1:400 in BB) plus AlexaFluor 555 donkey anti-mouse secondary antibody (1:400 in BB). Finally astrocytes were immunostained using a mouse anti-GFAP antibody conjugated with the fluorochrome AlexaFluor 488 (1:500 in BB).

#### 2.3.2 Abeta + IBA1 double labelling immunohistochemistry

*Day 1*: After BB, sections were incubated overnight at 4°C in a solution containing two primary antibodies: a mouse anti-Abeta antibody (1:200, in BB) plus a rabbit anti-IBA1 antibody (1:300, in BB).

*Day 2*: Sections were incubated with AlexaFluor 488 donkey anti-rabbit (1:400, in BB) and then with a solution of AlexaFluor 488 donkey anti-rabbit (1:400, in BB) plus AlexaFluor 555 donkey anti-mouse (1:400, in BB).

#### 2.3.3 NeuN + CD68 + IBA1 triple labelling immunohistochemistry

*Day 1*: After BB, sections were incubated overnight at 4°C with a solution containing three primary antibodies: a mouse anti-NeuN antibody (1:400, in BB) plus a rabbit anti-CD68 antibody (1:200, in BB) and a goat anti-IBA1 antibody (1:200, in BB).

*Day 2*: Sections were incubated with AlexaFluor 488 donkey anti-rabbit (1:400, in BB) and then with a solution of AlexaFluor 488 donkey anti-rabbit (1:400, in BB) plus AlexaFluor 635 donkey anti-goat (1:400, in BB). Finally, sections were incubated with a solution of AlexaFluor 488 donkey anti-rabbit (1:400, in BB) plus AlexaFluor 635 donkey anti-goat (1:400, in BB) plus AlexaFluor 555 donkey anti-mouse (1:400, in BB).

#### 2.3.4 CX3CR1 + IBA1 double labelling immunohistochemistry

*Day 1:* After BB, sections were incubated overnight at 4°C with a solution of two primary antibodies: a rabbit anti-CX3CR1 antibody (1:200, in BB) and a goat anti-IBA1 antibody (1:200, in BB).

*Day 2: S*ections were incubated with AlexaFluor 488 donkey anti-rabbit (1:400, in BB) and then with AlexaFluor 488 donkey anti-rabbit (1:400, in BB) plus AlexaFluor 635 donkey anti-goat (1:400, in BB).

### 2.4 Microscopy acquisition and images quantitative analyses

All sections were mounted onto gelatin-coated slides using Vectashield mounting medium with DAPI (Vectashield Product Code #H-1200, Vector, Burlingame, CA, United States) and then observed under a LEICA TCS SP8 confocal laser scanning microscope (Leica Microsystems CMS GmbH, Mannheim, Germany) equipped with 20X, 40X or 63X objective (z step: 1.2 µm, 0.6 µm or 0.3 µm respectively). Frequency of acquisition was 200 Hz and frame of acquisition was 1,024 pixels x 1,024 pixels. Confocal scans were acquired keeping all parameters constant.

Confocal acquisitions were performed in the Regions of Interest (ROI) CA1 and CA3 areas of the hippocampus (Lorente De Nó, 1934; Li et al., 1994). The CA1 and CA3 ROI were subdivided into two subregions Stratum Pyramidalis (SP) and Stratum Radiatum (SR) (Amaral and Lavenex, 2007; Lana et al., 2016). All quantitative analyses were performed on z-projection of 10 consecutive z confocal scans (20X objective, z step 1.2 µm, total thickness 12 µm) using the software ImageJ software (National Institute of Health, http://rsb.info.nih.gov/ij).

**FIGURE 1:**
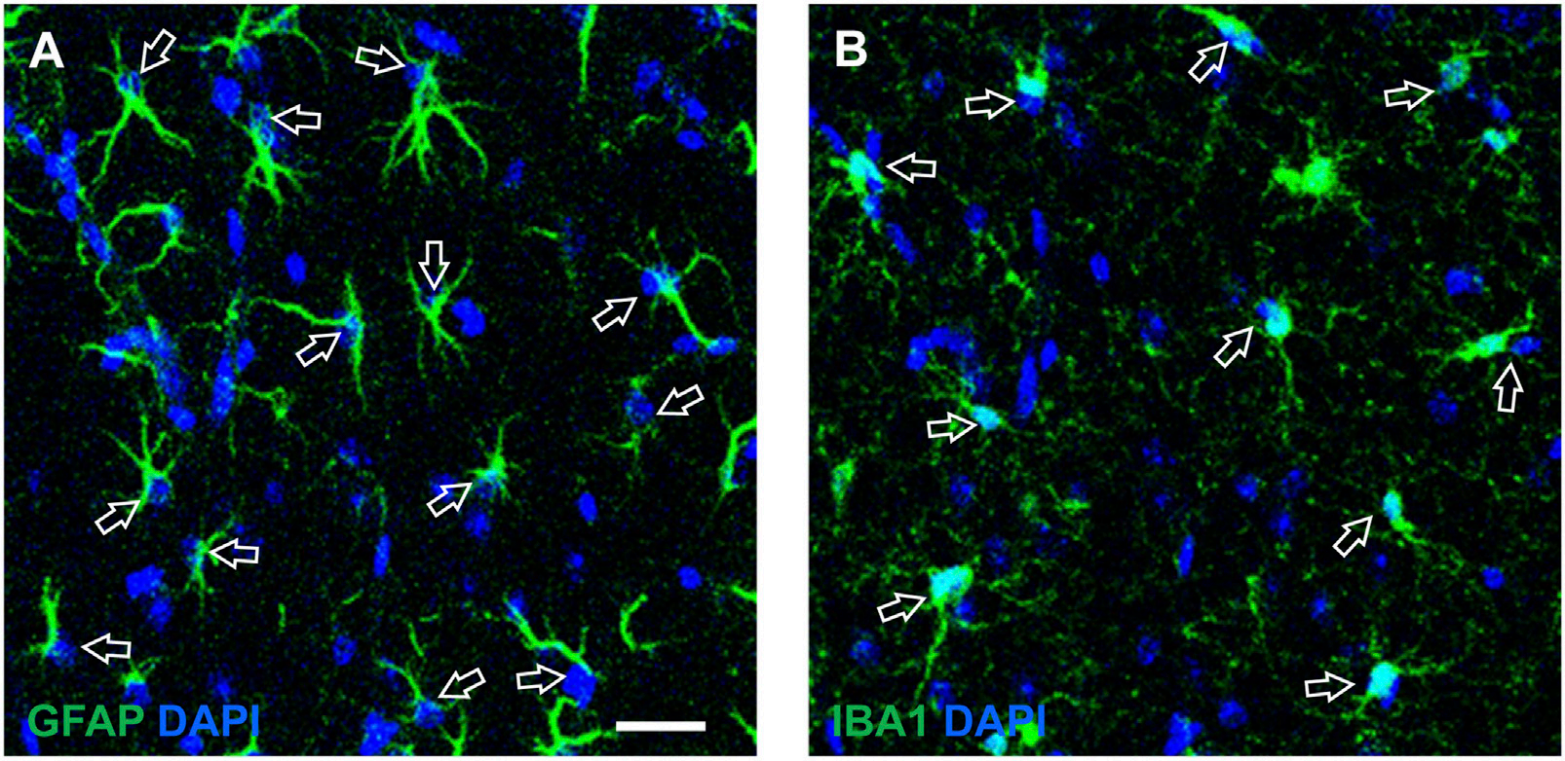
Enlargements of representative confocal images of CA1 SR exemplifying the quantitative analysis of astrocytes **(A)** and microglia **(B)**, counterstained with DAPI. The open arrows indicate astrocytes **(A)** and microglia **(B)** that can be easily recognized not only from their immunostaining and shape, but also from the presence of DAPI nuclear staining. **(A)** Each counted astrocyte was characterized by the colocalization of GFAP immunostaining (green) and DAPI staining (blue). **(B)** Each counted microglia cell was characterized by the colocalization of IBA1 immunostaining (green) and DAPI staining (blue). Scale bar: 50 μm **(A,B)**.

Density of neurons, beta amyloid plaques, astrocytes and microglia were expressed as plaques or cell/mm2. For the quantification of astrocytes and microglia we counted GFAP or IBA1 immunostained cells which were counterstained with DAPI, as exemplified by the arrows in Figures 1A, B. We evaluated the density of two population of microglia, total microglia and “ball-and-chain” microglia. The latter are characterized by spherical phagocytic pouches (ball) at the tip of microglial terminal branches (chain), the so-called ball-and-chain structures, which can phagocytose apoptotic debris or a small quantity of other substances (Sierra et al., 2010). Ball-and-chain microglia were included in the quantification if they had at least one pouch with dimensions above 10 μm, measured using the analyze tool of ImageJ.

Quantitative analyses of GFAP and IBA1 expression/cell was obtained from the percentage of GFAP + or IBA1 + pixels above a threshold level/number of astrocytes or microglia using the threshold tool on ImageJ (see Gerace et al., 2021).

To further characterize microglia cells population, we investigated reactive microglia with CD68 and CX3CR1 immunostaining. We quantified CD68 (or CX3CR1) expression/cell as total number of CD68+ (or CX3CR1+) pixels above a threshold level/number of CD68+ (or CX3CR1+) cell.

### 2.5 Statistical analysis

Data are presented as means ± SEM. Statistical significance were evaluated by Student’s t-test, one-way ANOVA followed by Newman-Keuls multiple comparison test or by two-way ANOVA followed by Bonferroni post test, as required. All statistical analyses were performed using GraphPad PRISM v. 5 for Windows (GraphPad Software, San Diego, CA, United States). A probability value (*P*) of <0.05 was considered significant.

## 3 Results

### 3.1 The pre- and probiotics enriched diet decreases Aβ plaques in CA3 but not in CA1 hippocampus of APP/PS1 mice

**FIGURE 2:**
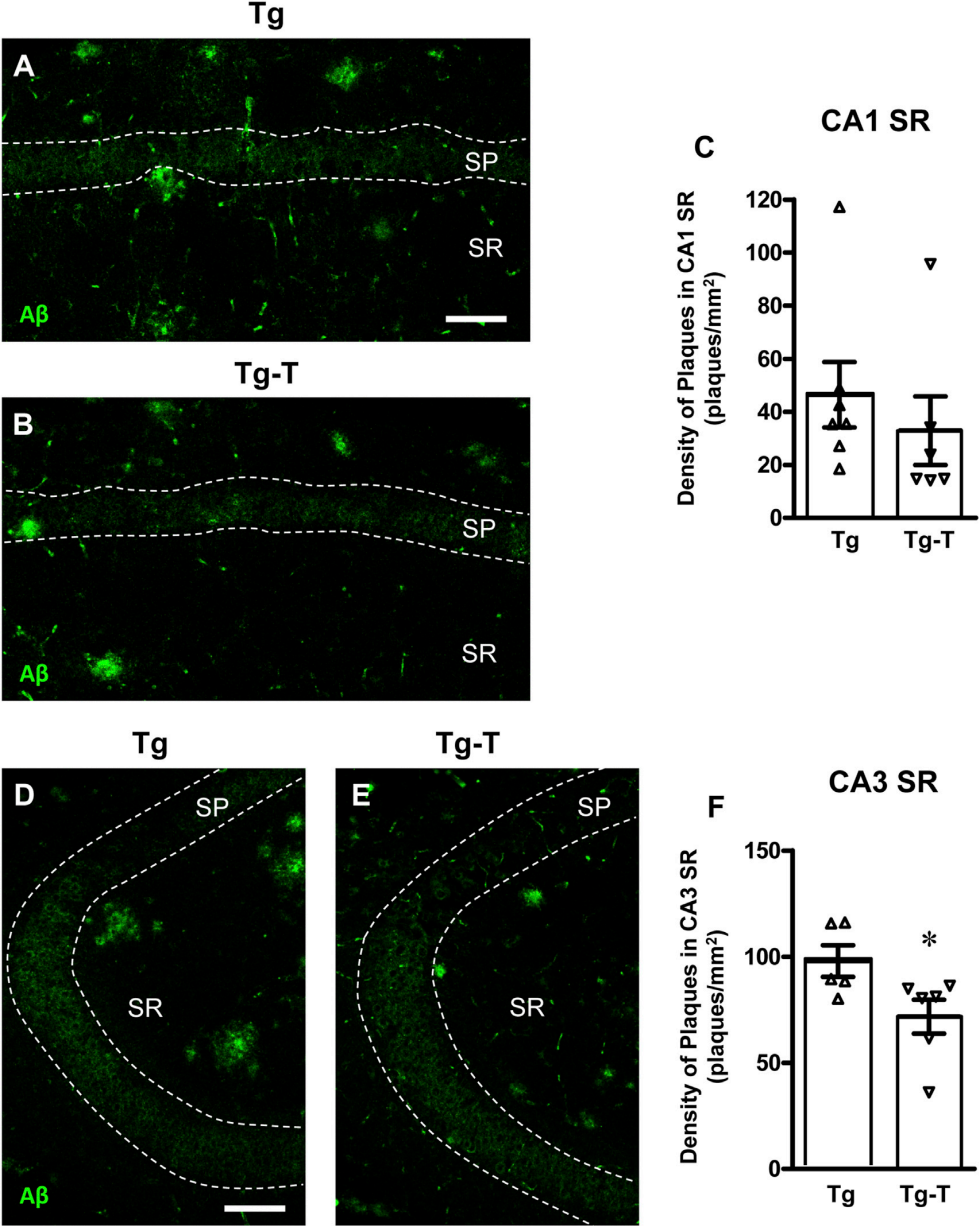
Analysis of Aβ plaques in CA1 and CA3 hippocampus of Tg and Tg-T mice. **(A,B)** Representative confocal images of Aβ immunostaining (green) in CA1 hippocampus of a Tg **(A)** and a Tg-T **(B)** mice. Scale bar: 50 μm. **(C)** Quantitative analysis of Aβ plaques/mm^2^ in CA1 Stratum Radiatum (SR) of Tg (n = 7) and Tg-T mice (n = 6; *P < 0.05 vs. Tg, Student’s t test). **(D,E)** Representative confocal images of Aβ immunostaining (green) in CA3 hippocampus of a Tg **(D)** and a Tg-T **(E)** mice. Scale bar: 50 μm. **(F)** Quantitative analysis of Aβ plaques/mm^2^ in CA3 SR of Tg (n = 5) and Tg-T mice (n = 6, *P* = 0.4668 vs. Tg, n.s., Student’s t test). Data reported in all graph bars are expressed as mean ± SEM.

Beta-amyloid plaques were immunolabelled using an anti-Aβ antibody in hippocampal sections of 8 months old APP/PS1 mice (Figures 2A, D, Tg) and of APP/PS1 mice fed with pre- and probiotics (Figures 2B, E, Tg-T). Immunostaining in CA1 (Figures 2A, B) and CA3 (Figures 2D, E) hippocampal areas were acquired by confocal microscopy. Quantitative analyses of Aβ plaques density in the Stratum Radiatum (SR) of CA1 (Figure 2C) and CA3 areas (Figure 2F) demonstrated that Aβ plaques density (plaques/mm^2^) was significantly lower in CA3 of Tg-T (−27%) than in CA3 of Tg. The density of Aβ plaques was not significantly different in CA1 of Tg-T mice in comparison with Tg mice (−29%).

### 3.2 The pre- and probiotics enriched diet protected neurons in CA1 hippocampus of APP/PS1 mice

**FIGURE 3:**
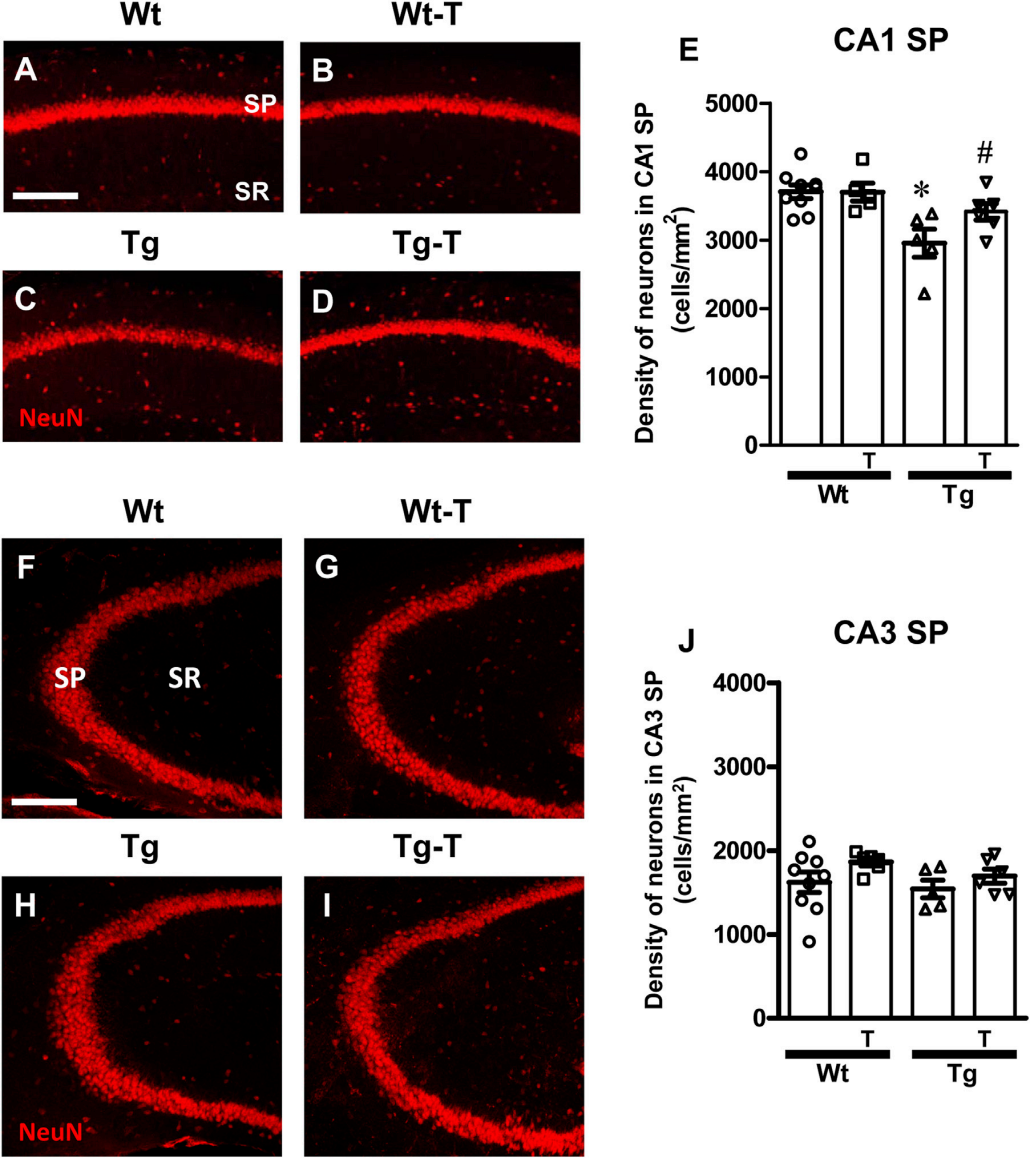
Analysis of neurons in CA1 and CA3 areas of Wt, Wt-T, TG and Tg-T mice. **(A–D)** Representative confocal images of NeuN immunostaining of neurons (red) in CA1 of Wt **(A)**, Wt-T **(B)**, Tg **(C)** and Tg-T **(D)** mice. Scale bar: 100 μm. **(E)** Quantitative analysis of neurons/mm^2^ in CA1 Stratum Pyramidale (SP) of Wt (n = 9), Wt-T (n = 5), Tg (n = 5) and Tg-T mice (n = 6); Statistical analysis: One-way ANOVA: F (3, 24) = 6.307, *P* = 0.0032; Newman-Keuls post-test: **P < 0.01 Tg vs. Wt, and vs. Wt-T; *P < 0.05 Tg vs. Tg-T. **(F–I)** Representative confocal images of NeuN immunostaining of neurons (red) in CA3 of Wt **(F)**, Wt-T **(G)**, Tg **(H)** and Tg-T **(I)** mice. Scale bar: 100 μm. **(J)** Quantitative analysis of neurons/mm^2^ in CA3 SP of Wt (n = 9), Wt-T (n = 6), Tg (n = 5) and Tg-T mice (n = 6). Statistical analysis: One-way ANOVA: F (3, 25) = 1.563, *P* = 0.2265, not significant. Data reported in all graph bars are expressed as mean ± SEM.

We evaluated the density of CA1 and CA3 pyramidal neurons using immunohistochemical staining with anti-NeuN antibody (Figure 3, red) in hippocampal sections of the four experimental groups. Representative images of NeuN immunostaining in CA1 (Figures 3A–D) and CA3 (Figures 3F–I) hippocampal areas were acquired by confocal microscopy, and quantitative analyses of neuronal density in the Stratum Pyramidalis (SP) of CA1 (Figure 3E) and CA3 areas (Figure 3J) were performed.

Quantitative analyses shown in the graphs (Figures 3E, J for CA1 and CA3, respectively) demonstrated that the density of neurons in CA1 SP of Tg mice was significantly lower than in CA1 of Wt mice (−20% compared with Wt, Figure 3E). In Tg-T mice, we found that the density of CA1 SP neurons was significantly higher than in Tg mice, and not statistically different from that of Wt mice. The decreased degeneration of neurons (−8%) indicates that the treatment has a protective effect.

In the CA3 region of Tg mice, the density of SP neurons was not significantly different among the four experimental groups, as demonstrated by statistical analysis (Figure 3J).

### 3.3 The pre- and probiotics enriched diet modified astrocytes activation in CA1 and CA3 hippocampus of APP/PS1 mice

**FIGURE 4:**
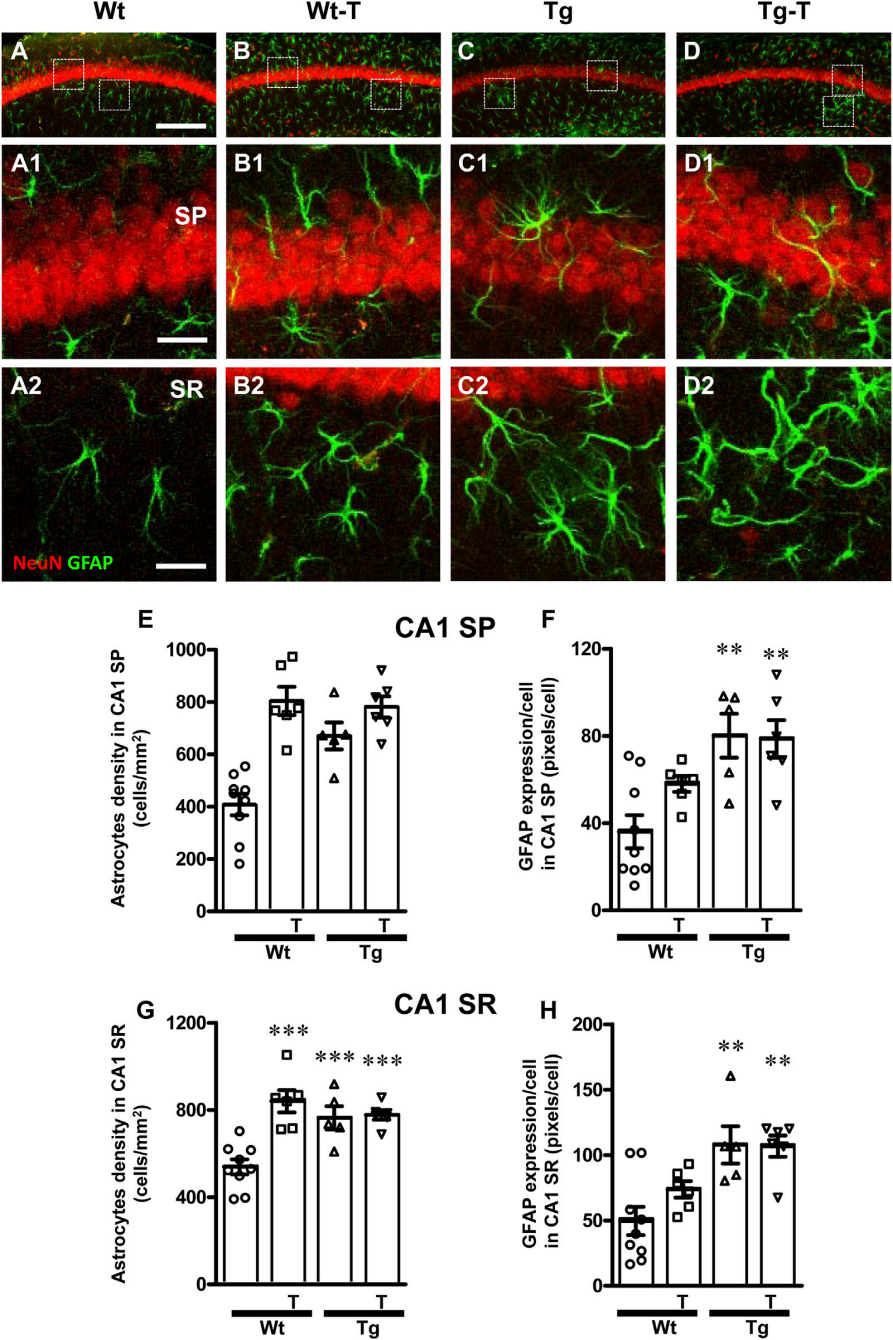
Analysis of astrocytes in CA1 areas of Wt, Wt-T, Tg and Tg-T mice. **(A–D1)** Representative confocal images of GFAP immunostaining of astrocytes (green) and neurons (red) in CA1 of a Wt (A,A1), a Wt-T (B,B1), a Tg (C,C1) and a Tg-T (D,D1) mice. Scale bar: 100 μm **(A–D)**. **(A1–D1)** magnifications of the framed areas in the corresponding panels in SP above. Scale bar: 20 μm. **(A2–D2)** magnifications of the framed areas in the corresponding panels in SR above. Scale bar: 20 µm. **(E,G)** Quantitative analysis of astrocyte density (cells/mm^2^) in the CA3 SP **(E)** and SR **(G)** of Wt (n = 9), Wt-T (n = 6), Tg (n = 5) and Tg-T mice (n = 6). **(E)** Statistical analysis: One-way ANOVA: F (3, 25) = 17.97, *P* < 0.0001; Newman-Keuls post-test: ***P < 0.01 Wt vs. all other groups). **(G)** Statistical analysis: One-way ANOVA: F (3, 25) = 12.75, *P* < 0.0001; Newman-Keuls post-test: ***P < 0.01 Wt vs. all other groups. **(F,H)** Quantitative analysis of GFAP expression per astrocyte (% positive pixels/cell) in CA1 SP **(F)** and SR **(H)** of Wt (n = 9), Wt-T (n = 6), Tg (n = 5) and Tg-T mice (n = 6). **(F)** Statistical analysis: One-way ANOVA: F (3, 25) = 7.703, *P* < 0.001; Newman-Keuls post-test: **P < 0.01 Wt vs. Tg and Wt vs. Tg-T). **(H)** Statistical analysis: One-way ANOVA: F (3, 25) = 7.8, *P* < 0.001; Newman-Keuls post-test: **P < 0.01 Wt vs. Tg and Wt vs. Tg-T. Data reported in all graph bars are expressed as mean ± SEM.

**FIGURE 5:**
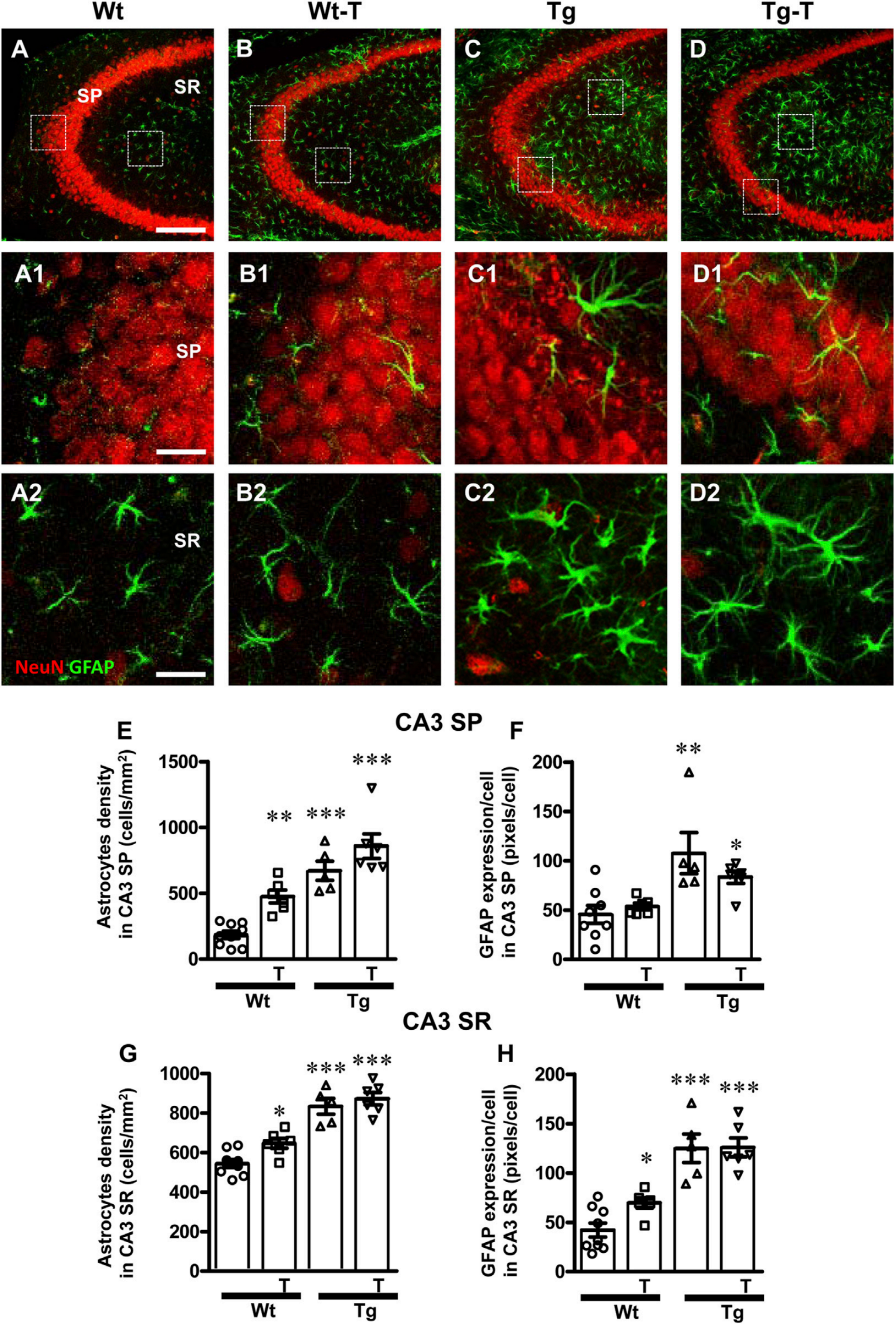
Analysis of astrocytes in CA3 areas of Wt, Wt-T, Tg and Tg-T mice. **(A–D1)** Representative confocal images of GFAP immunostaining of astrocytes (green) and neurons (red) in CA3 of a Wt (A,A1), a Wt-T (B,B1), a Tg (C,C1) and a Tg-T (D,D1) mice. Scale bar: 100 μm **(A–D)**. **(A1–D1)** magnifications of the framed areas in the corresponding panels in SP above. Scale bar: 20 μm. **(A2–D2)** magnifications of the framed areas in the corresponding panels in SP above. Scale bar: 20 µm. **(E,G)** Quantitative analysis of astrocyte density (cells/mm^2^) in the CA3 SP **(E)** and SR **(G)** of Wt (n = 9), Wt-T (n = 6), Tg (n = 5) and Tg-T mice (n = 6). **(E)** Statistical analysis: One-way ANOVA: F (3, 25) = 26.61, *P* < 0.0001; Newman-Keuls post-test: ***P < 0.001 Wt vs. Tg and Tg-T; **P < 0.01 Wt-T vs. Wt). **(G)** Statistical analysis: One-way ANOVA: F (3, 25) = 23.03, *P* < 0.0001; Newman-Keuls post-test: *P < 0.05 Wt vs. Wt-T; ***P < 0.001 Wt vs. Tg and Tg-T. **(F)** Quantitative analysis of GFAP expression per astrocyte (% positive pixels/cell) in CA3 SP **(F)** of Wt (n = 8), Wt-T (n = 6), Tg (n = 5) and Tg-T mice (n = 6). Statistical analysis: One-way ANOVA: F (3, 24) = 6.826, *P* < 0.01; Newman-Keuls post-test: **P < 0.01 Wt vs. Tg; *P < 0.05 Wt vs. Tg-T. **(H)** Quantitative analysis of GFAP expression per astrocyte (% positive pixels/cell) in CA3 SR of Wt (n = 9), Wt-T (n = 6), Tg (n = 5) and Tg-T mice (n = 6). Statistical analysis: One-way ANOVA: F (3, 25) = 23.03, *P* < 0.0001; Newman-Keuls post-test: *P < 0.05 Wt vs. Wt-T; ***P < 0.001 Wt vs. Tg and Tg-T. Data reported in all graph bars are expressed as mean ± SEM.

Astrocytes were visualized by immunohistochemistry using an anti-GFAP antibody (Figures 4, 5, green) in CA1 and CA3 hippocampal sections of of the four experimental groups. Neurons were counterstained with anti NeuN antibody (Figures 4, 5). Images of fluorescent immunostaining were taken separately in CA1 (Figures 4A–D2) and CA3 hippocampal regions (Figures 5A–D2) with confocal microscopy and qualitative and quantitative analyses were performed separately in CA1 and CA3 SP and SR. From the representative confocal images, it is apparent that astrocytes are more numerous and more reactive in the hippocampus of both Tg and Tg-T mice, and are localized especially around plaques (See Figures 9F, F1, G, H).

The graphs in Figure 4 show the density of astrocytes and GFAP expression/astrocyte in CA1 SP (Figures 4E, F) and CA1 SR (Figures 4G, H) of the four experimental groups. Quantitative analysis performed in CA1 SP showed that astrocytes density increased significantly in CA1 SP of Tg mice and in both Wt-T and Tg-T mice in comparison to Wt mice (Figure 4E, Wt-T + 97% vs. Wt; Tg + 64% vs. Wt; Tg-T + 91% vs. Wt). The expression of GFAP/cell, as a marker of astrocyte activation, increased in CA1 SP of Tg and Tg-T mice vs. Wt mice (Figure 4F, Tg + 122% vs. Wt; Tg-T + 119% vs. Wt).

Quantitative analysis performed in CA1 SR showed that astrocytes density increased significantly in the CA1 SR of Tg mice and in both Wt-T and Tg-T mice (Figure 4G, Wt-T + 56% vs. Wt; Tg + 42% vs. Wt; Tg-T + 44% vs. Wt). The expression of GFAP/cell increased in CA1 SR of both Tg and Tg-T vs. Wt mice (Figure 4H, Tg + 116% vs. Wt; Tg-T 114% vs. Wt).

The graphs in Figure 5 show astrocytes density and GFAP expression/astrocyte in CA3 SP (Figures 5E, F) and CA3 SR (Figures 5G, H) of the four experimental groups. Quantitative analysis performed in CA3 SP showed that astrocytes density increased significantly in the CA3 SP of Wt-T mice, and, even more consistently, in both Tg and Tg-T mice in comparison to Wt mice (Figure 5E, Wt-T + 157% vs. Wt; Tg + 263% vs. Wt; Tg-T + 364% vs. Wt). The expression of GFAP/cell, as a marker of astrocyte activation, increased significantly in CA1 SP of Tg mice, but in CA3 SP of Tg-T the effect was less intense, although still significant in comparison to Wt mice (Figure 5F, Tg + 135% vs. Wt; Tg-T + 80% vs. Wt).

Quantitative analysis performed in CA3 SR showed that astrocytes density increased significantly in the CA3 SR of Wt-T, Tg and Tg-T mice in comparison with Wt mice (Figure 5G, Wt-T + 19% vs. Wt; Tg + 53% vs. Wt; Tg-T + 60% vs. Wt). The expression of GFAP/cell increased in CA3 SR of both Wt-T, Tg and Tg-T mice vs. Wt mice (Figure 5H, Wt-T + 67% vs. Wt; Tg + 198% vs. Wt; Tg-T + 200% vs. Wt).

### 3.4 The pre- and probiotics enriched diet modified microglia reactivity in CA1 and CA3 hippocampus of APP/PS1 mice

**FIGURE 6:**
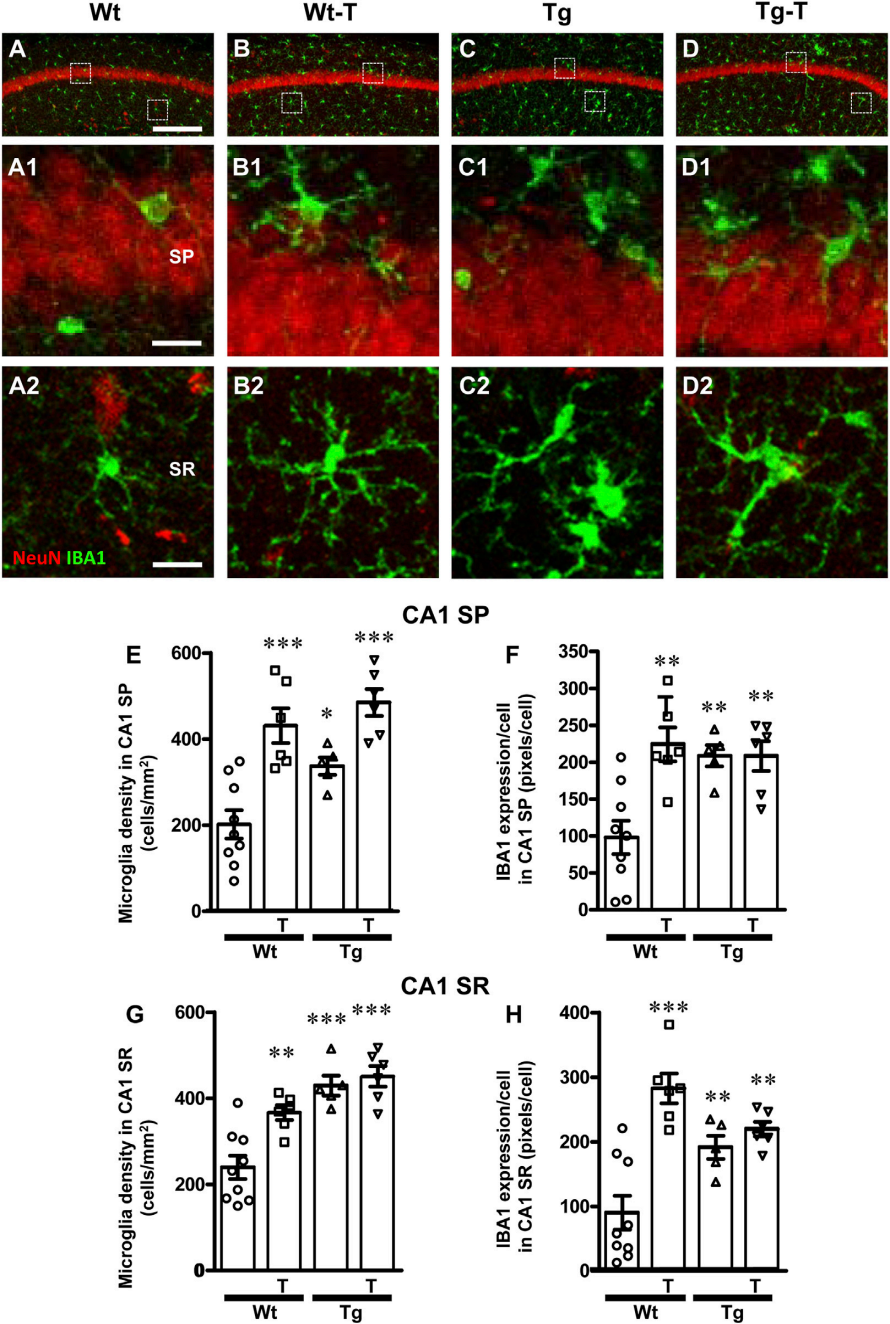
Analysis of total microglia in CA1 areas of Wt, Wt–T, Tg and Tg-T mice. **(A–D1)** Representative confocal images of IBA1 immunostaining of microglia (green) and neurons (red) in CA1 of a Wt (A,A1), a Wt-T (B,B1), a Tg (C,C1) and a Tg-T (D,D1) mouse. Scale bar: 100 μm **(A–D)**. **(A1–D1)** magnifications of the framed areas in the corresponding panels in SP above. Scale bar: 20 μm. **(A2–D2)** magnifications of the framed areas in the corresponding panels in SR above. Scale bar: 20 µm. **(E,G)** Quantitative analysis of microglia density (cells/mm^2^) in the CA1 SP **(E)** and SR **(G)** of Wt (n = 9), Wt-T (n = 6), Tg (n = 5) and Tg-T mice (n = 6). **(E)** Statistical analysis: One-way ANOVA: F (3, 25) = 15.45, *P* < 0.0001; Newman-Keuls post-test: ***P < 0.001 Wt-T and Tg-T vs. Wt; *P < 0.05 Tg vs. Wt. **(G)** Statistical analysis: One-way ANOVA: F (3, 25) = 16.48, *P*< 0.0001; Newman-Keuls post-test: **P < 0.01 Wt vs. Wt–T, ***P < 0.001 Tg and Tg–T vs. Wt. **(F,H)** Quantitative analysis of IBA1 expression per microglia (% positive pixels/cell) in CA1 SP **(F)** and SR **(H)** of Wt (n = 9), Wt-T (n = 6), Tg (n = 5) and Tg-T mice (n = 6). **(F)** Statistical analysis: One-way ANOVA: F (3, 25) = 8.498, *P* < 0.001; Newman-Keuls post-test: **P < 0.01 Wt vs. all other groups). **(H)** Statistical analysis: One-way ANOVA: F (3, 25) = 14.14, *P* < 0.001; Newman-Keuls post-test: ***P < 0.001 Wt-T vs. Wt, **P < 0.01 Tg and Tg-T vs. Wt. Data reported in all graph bars are expressed as mean ± SEM.

**FIGURE 7:**
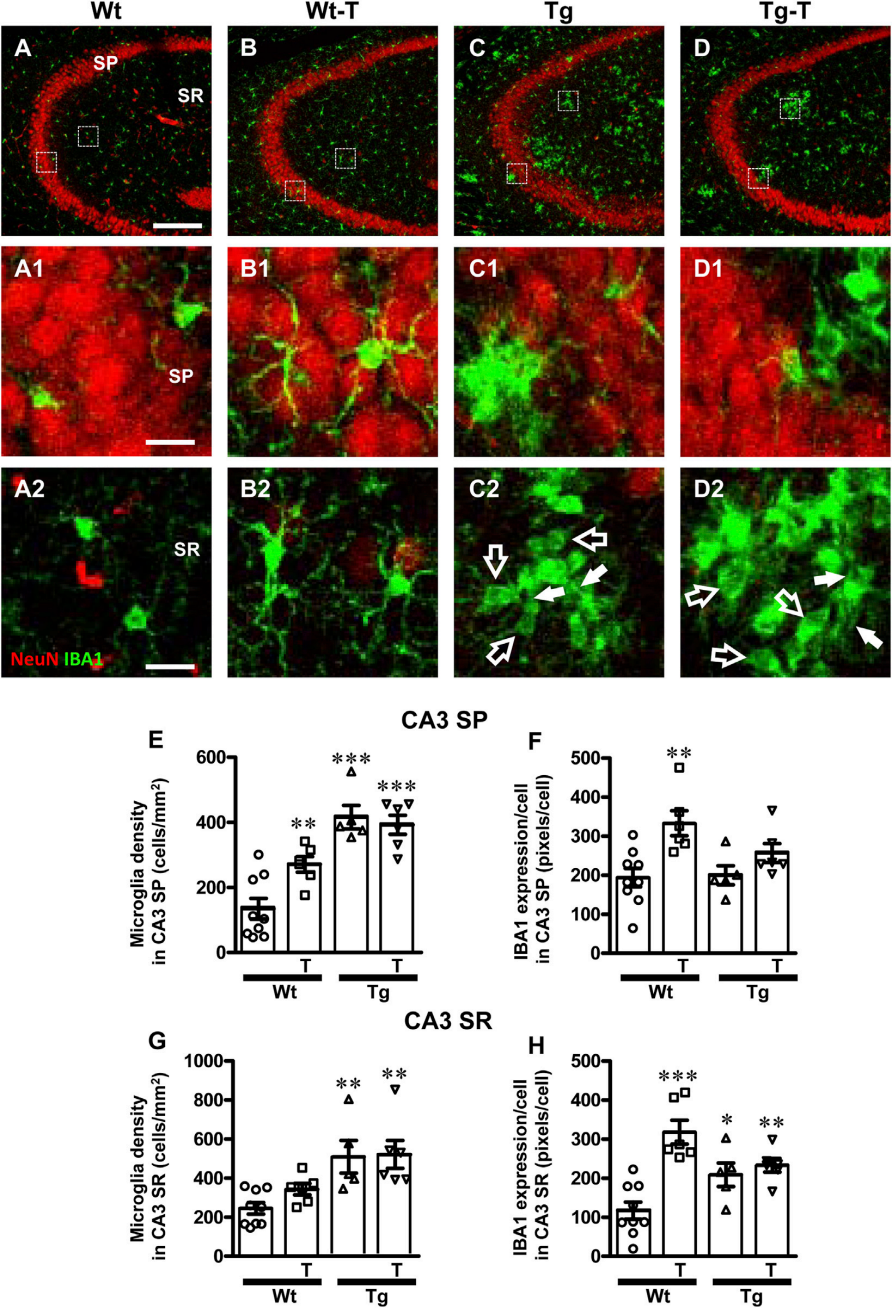
Analysis of total microglia in CA3 areas of Wt, Wt-T, Tg and Tg-T mice. **A–D1** Representative confocal images of IBA1 immunostaining of microglia (green) and neurons (red) in CA3 of a Wt (A,A1), a Wt-T (B,B1), a Tg (C,C1) and a Tg-T (D,D1) mouse. Scale bar: 100 μm **(A–D)**. **(A1–D1)** magnifications of the framed areas in the corresponding panels in SP above. Scale bar: 20 μm. **(A2–D2)** magnifications of the framed areas in the corresponding panels above, showing the presence of ball-and-chain microglia in Tg and Tg-T CA3 SR (spherical phagocytic pouches are indicated by open arrows, microglia branches by white arrows). Scale bar: 20 µm. **(E,G)** Quantitative analysis of microglia density (cells/mm^2^) in the CA3 SP **(E)** and SR **(G)** of Wt (n = 9), Wt-T (n = 6), Tg (n = 5) and Tg-T mice (n = 6). **(E)** Statistical analysis: One-way ANOVA: F (3, 25) = 18.66, *P* < 0.0001; Newman-Keuls post-test: ***P < 0.001 Tg and Tg-T vs. Wt;, **P < 0.01 Wt-T vs. Wt. **(G)** Statistical analysis: One-way ANOVA: F (3, 25) = 7.187, *P*< 0.001; Newman-Keuls post-test: **P < 0.01 Tg and Tg-T vs. Wt. **(F,H)** Quantitative analysis of IBA1 expression per microglia (% positive pixels/cell) in CA3 SP **(F)** and SR **(H)** of Wt (n = 9), Wt-T (n = 6), Tg (n = 5) and Tg-T mice (n = 6). **(F)** Statistical analysis: One-way ANOVA: F (3, 25) = 5.931, *P* < 0.01; Newman-Keuls post-test: **P < 0.01 Wt-T vs. Wt. **(H)** Statistical analysis: One-way ANOVA: F (3, 25) = 12.28, *P* < 0.0001; Newman-Keuls post-test: ***P < 0.001 Wt-T vs. Wt, *P < 0.05 Tg vs. Wt, **P < 0.01 Tg-T vs. Wt. Data reported in all graph bars are expressed as mean ± SEM.

**FIGURE 8:**
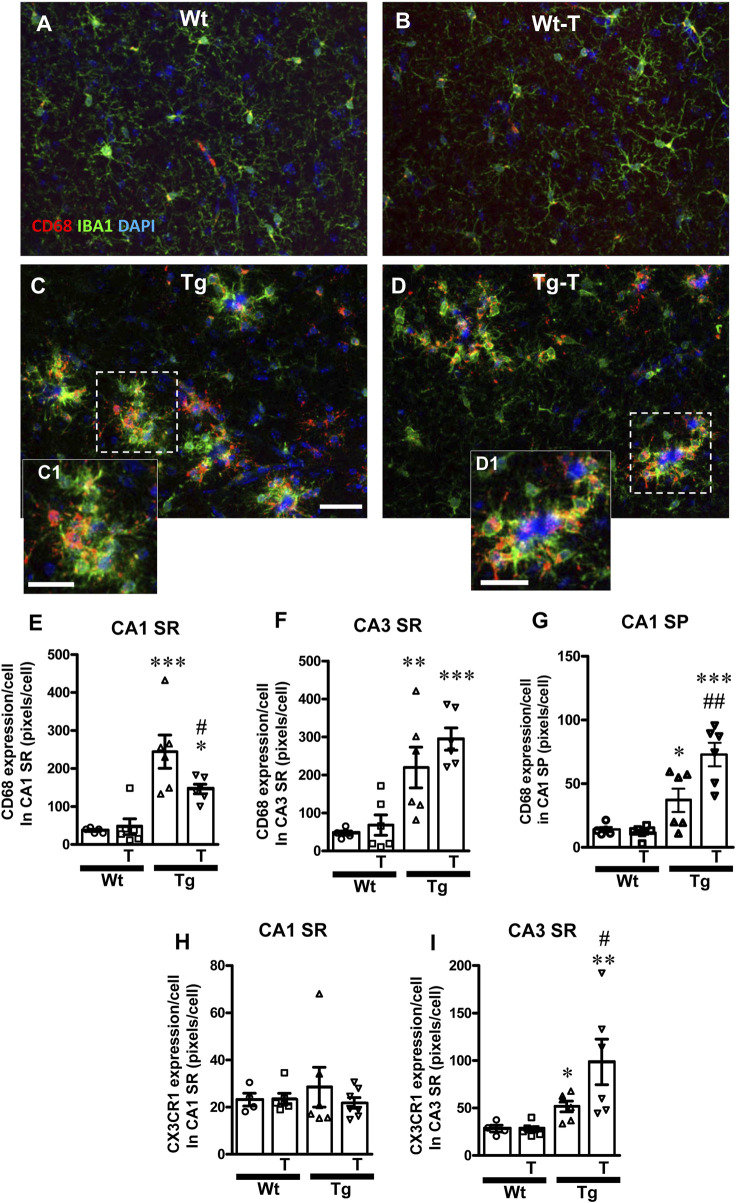
Analysis of ball-and-chain microglia in CA1 and CA3 SR of Wt, Wt-T, Tg and Tg-T mice. **(A–D)** Representative confocal images of immunostaining of microglia (IBA1, green and CD68, red) counterstained for nuclei with DAPI (blue) in CA3 SR of Wt **(A)**, a Wt-T **(B)**, a Tg **(C)** and a Tg-T **(D)** mice. Scale bar: 20 μm **(A–D)**. **(C1,D1)** magnifications of the framed areas in the corresponding panels above, showing the coocalization of CD68 with IBA1. Scale bars 12 µm. **(E,F)** Quantitative analysis of CD68 expression/cell in CA1 SR **(E)** and CA3 SR **(F)** of Wt (n = 5), Wt-T (n = 6), Tg (n = 6) and Tg-T mice (n = 6). **(E)** Statistical analysis: One-way ANOVA: F (3, 22) = 13.66, *P* < 0.0001; Newman-Keuls post-test: ***P < 0.001 Tg vs. Wt, *P < 0.01 Tg-T vs. Wt, #*P* < 0.05 Tg-T vs. Tg. **(F)** Statistical analysis: One-way ANOVA: F (3, 22) = 11.53, *P* < 0.001; Newman-Keuls post-test: ***P < 0.001 Tg-T vs. Wt, **P < 0.01 Tg vs. Wt. **(G)** Quantitative analysis of CD68 expression/cell in CA1 SP of Wt (n = 5), Wt-T (n = 6), Tg (n = 6) and Tg-T mice (n = 6). Statistical analysis: One-way ANOVA: F (3, 22) = 16.94, *P* < 0.0001; Newman-Keuls post-test: ***P < 0.001 Tg-T vs. Wt, **P < 0.01 Tg-T vs. Tg, *P < 0.05 Tg vs. Wt. **(H,I)** Quantitative analysis of CX3CR1 expression/cell in CA1 SR **(H)** and CA3 SR **(I)** of Wt (n = 4), Wt-T (n = 6), Tg (n = 6) and Tg-T mice (n = 7). **(H)** Statistical analysis: One-way ANOVA: F (3, 22) = 0.3835, n.s. **(I)** Statistical analysis: One-way ANOVA: F (3,21) = 6.085, *P* < 0.01; Newman-Keuls post-test: **P < 0.01 Tg-T vs. Wt, *P < 0.05 Tg vs. Wt.

**FIGURE 9:**
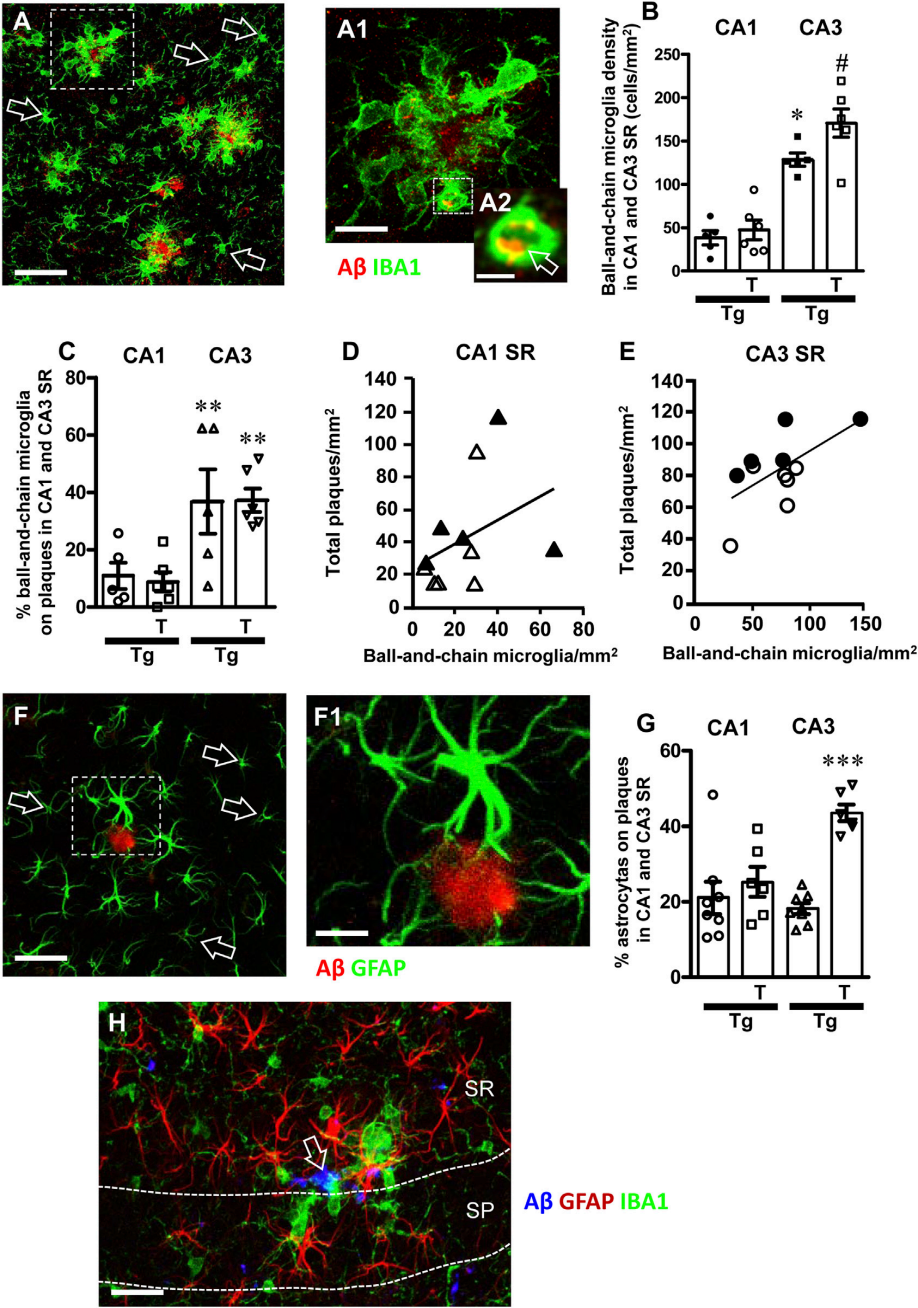
**(A)** Representative confocal image of microglia (IBA1, green) and beta-amyloid (red) in CA3 SR of a Tg-T mouse to evidence the different phenotypes of ball-and-chain microglia (green) around beta-amyloid plaques (red) and far from the plaques (open arrows). Scale bar: 50 μm. **(A1)** Enlargement of the cluster of ball-and-chain microglia (IBA1, green) around a beta-amyloid plaque (red), from the framed area in panel **(A)**. Total thickness of the confocal stack: 20 µm. Scale bar: 10 μm. **(A2)** Enlargement of the ball-and chain pouch in A1 to evidence the phagocytosis of beta-amyloid (yellow-orange color pointed out by the open arrow). Total thickness of the confocal stack: 1.5 µm. Scale bar: 5 μm. **(B)** Quantitative analysis of ball-and-chain microglia density (cells/mm^2^) in CA1 and CA3 SR of Tg (n = 5) and Tg-T mice (n = 6). Statistical analysis: two-way ANOVA with Area and Treatment as the two variables: significant main effect for Treatment, F (1, 18) = 5.51, *P* = 0.0029, Area, F (1, 18) = 82.65, *P* < 0.001, and Interaction Treatment-Area: F (1, 18) = 3.46, *P* = 0.0138. **(C)** Quantitative analysis of the percentage of ball and chain microglia on plaques in CA1 and CA3 SR of Tg (n = 5) and Tg-T mice (n = 6). Statistical analysis: two-way ANOVA with Area and Treatment as the two variables: significant main effect for Treatment, F (1, 18) = 0.01651, *P* = 0.0165, Area, F (1, 18) = 19.32, *P* = 0.0003, and Interaction Treatment-Area: F (1, 18) = 0.0438, *P* = 0.8366. **P < 0.01 Tg CA3 vs. Tg CA1 and Tg-T CA3 vs. Tg T CA1. **(D,E)** Correlation of ball-and-chain microglia density with beta-amyloid plaques density in CA1 SR **(D)** (n = 10) and CA3 SR **(E)** (n = 11). Black triangles: Tg CA1 SR; White triangles: Tg-T CA1 SR; Black circles: Tg CA3 SR; White circles: Tg-T CA3 SR. **(F)** Representative confocal image of astrocytes (GFAP, green) and beta-amyloid (red) in CA3 SR of a Tg-T mouse to evidence the different phenotypes of astrocytes (green) around beta-amyloid plaques (red) and far from the plaques (open arrows). Scale bar: 40 μm. **(F1)** Enlargement of one astrocyte (GFAP, green) around a beta-amyloid plaque (red), from the framed area in panel **(A)**. Total thickness of the confocal stack: 20 µm. Scale bar: 20 μm. **(G)** Quantitative analysis of the percent of astrocytes surrounding Aβ plaques in CA1 and CA3 SR of Tg (n = 5) and Tg-T mice (n = 6). ***P < 0.001 Tg-T vs. all other groups. **(H)** Representative confocal image of microglia (IBA1, red), astrocytes (GFAP, green) and Aβ (blue) in CA3 of a Tg-T mouse at the limit of CA4, taken at the boundaries between SP and SR. Thickness of the confocal stacks: 15 µm. Scale bar: 10 µm.

To perform the characterization of microglia on hippocampal sections of the four experimental groups, microglia were immunolabelled with anti-IBA1 antibody in CA1 (Figures 6A–D2) and CA3 (Figures 7A1–D2) and reactive microglia with CD68 antibody (Figures 8A–D). Images of fluorescent immunostaining were taken in SP and SR of CA1 and CA3 hippocampal areas with confocal microscopy. The quantitative analyses of microglia were performed in CA1 and CA3 SP and SR, separately. From the representative images shown in Figures 6A1–D2, 7A1–D2, it is apparent that microglia changed their morphological features in CA3, and, to a lesser extent in CA1, especially around amyloid plaques (see Figures 9A, H).

The density of microglia increased significantly in CA1 SP of Wt-T, Tg and Tg-T mice in comparison to Wt mice (Figure 6E, Wt-T + 113% vs. Wt; Tg + 66% vs. Wt; Tg-T + 140% vs. Wt). The expression of IBA1/cell increased in CA1 SP of Wt-T, Tg and Tg-T mice vs. Wt mice (Figure 6F, Wt-T + 129% vs. Wt; Tg + 113% vs. Wt; Tg-T + 112% vs. Wt).

Quantitative analysis showed that microglia density increased significantly in the CA1 SR of Wt-T, Tg and Tg-T mice (Figure 6G, Wt-T + 53% vs. Wt; Tg + 78% vs. Wt; Tg-T + 87% vs. Wt). The expression of IBA1/cell increased in CA1 SR of Wt-T, Tg and Tg-T mice in comparison to Wt mice (Figure 6H, Wt-T + 213% vs. Wt; Tg + 112% vs. Wt; Tg-T + 143% vs. Wt).

The density of microglia increased significantly in CA3 SP of Wt-T, Tg and Tg-T mice in comparison to Wt mice (Figure 7E, Wt-T + 102% vs. Wt; Tg + 210% vs. Wt; Tg-T + 193% vs. Wt. The expression of IBA1/cell increased in CA3 SP of Wt-T mice vs. Wt mice (Figure 7F, Wt-T + 72% vs. Wt).

Quantitative analysis showed that microglia density increased significantly in the CA3 SR of Tg and Tg-T mice in comparison to Wt mice (Figure 7G, Tg + 108% vs. Wt; Tg-T + 113% vs. Wt). Quantitative analysis showed that expression of IBA1/cell increased significantly in the CA3 SR of Wt-T, Tg and Tg-T mice (Figure 7H, Wt-T + 172% vs. Wt; Tg + 78% vs. Wt; Tg-T + 99% vs. Wt).

Furthermore, from the qualitative analyses shown in Figures 7A1–D2, it is apparent that in CA3 SR of Tg and Tg-T mice microglia underwent a phenomic modification forming the so-called ball-and-chain cells (Sierra et al., 2010) characterized by the presence of spherical phagocytic pouches (ball, open arrows in Figures 7C2, 7D2) at the tip of microglia terminal branches (chain, arrows).

A further carachterization of ball-and chain microglia was performed. Microglia were immunostained with IBA1 (green), and CD68 (red) and nuclei were counterstained with DAPI (blue) in both CA1 and CA3 (Figures 8A–D). It is evident from the qualitative confocal images in Figures 8A–D that ball-and-chain microglia were not present in CA3 SR of Wt and Wt-T mice. On the contrary, Figures 8C, C1, 8D, D1 show the presence of many ball-and-chain microglia in CA3 SR of Tg and Tg-T mice, respectively. In CA3 SR of Tg and Tg-T mice the ball-and-chain microglia were positive for CD68 immunostaining (evidenced by the yellow-orange colour in Figures 8C, C1, 8D, D1), an indication that they were in a reactive state. Quantitative analyses of microglia activation, defined as CD68-positive pixels/microglia in CA1 SR and CA3 SR are shown in Figures 8E, F, respectively. In CA1 SR of Tg mice, microglia activation was significantly higher than in Wt and Wt-T mice, and was decreased significantly by the treatment (Figure 8E: Tg + 559% vs. Wt; Tg-T + 295% vs. Wt). Furthermore, quantitative analyses of CD68-positive pixels/microglia showed that in CA1 SP of Tg mice, microglia activation was significantly higher than in Wt and Wt-T mice (Figure 8G: Tg + 217% vs. Wt; Tg-T + 524% vs. Wt), and was increased significantly by the treatment (Figure 8G: Tg-T + 97% vs. Tg). In addition, in CA3 SR of Tg mice, microglia activation was significantly higher than in Wt and Wt-T mice, and it was further increased by the treatment (Figure 8F: Tg + 368% vs. Wt; Tg-T + 526% vs. Wt).

We analyzed the expression of the fraktalkine receptor, CX3CR1, and we found that it did not change significantly in CA1 SR of any of the experimantal groups (Figure 8H). In CA3 SR of Tg mice, CX3CR1 expression was significantly higher than in Wt and Wt-T mice, and it was further increased by the treatment (Figure 8I: Tg + 83% vs. Wt; Tg-T + 249% vs. Wt; Tg-T + 42% vs. Tg).

It is interesting to note that in CA3 SR of Tg and Tg-T mice, ball-and-chain microglia were disposed around plaques, as demonstrated in the qualitative confocal image shown in Figures 9A–A2 (green, immunostaining for IBA1, red immunostaining for Abeta), while far from the plaques the phenotype of microglia (open arrows) was not different from those of Wt or Wt-T mice. Indeed, in CA1 or CA3 SR of Wt and Wt-T mice ball-and-chain microglia were extremely rare (Figure 9A), as also evinced by the quantitative analysis (Figure 9B). The quantitative analyses, performed in CA1 SR and CA3 SR of Tg and Tg-T mice, demonstrated that in both Tg and Tg-T mice, the density of ball-and-chain microglia in CA1 SR was very low and it was significantly lower than in CA3 SR (Figure 9B). Furthermore, in CA1 SR there was no significant difference between Tg and Tg-T mice. In CA3 SR the treatment increased significantly ball-and-chain microglia (Tg-T + 34% vs. Tg). Statistical analysis carried out by two-way ANOVA with Area and Treatment as the two variables revealed that there was a significant main effect for Treatment, F (1, 18) = 5.51, *P* = 0.0029, Area, F (1, 18) = 82.65, *P* < 0.001, and Interaction Treatment-Area: F (1,18) = 3.46, *P* = 0.0138. Bonferroni post test showed that there was a significant difference between areas (*P* < 0.001, CA3 vs. CA1), and treatment with pre-and pro-biotics significantly increased ball-and chain formation in CA3 SR only (*P* < 0.001, Tg-T vs. Tg).

The spherical pouches of ball-and-chain microglia (Sierra et al., 2010) can phagocytose small quantity of substances such as beta-amyloid. Figure 9A1 shows the enlargement of the framed area in Figure 9A. Figure 9A2 is the z-stack of five consecutive confocal scans for a total thickness of 1.5 µm, taken in the depth of the pouch of a ball-and-chain microglia. The yellow-orange colour evidenced by the open arrow indicates that beta-amyloid (red) is being phagocytosed by the microglia cell (green).

The density of ball and chain microglia in CA1 and CA3 SR shown in Figure 9B was correlated with the density of amyloid plaques quantified in the same area, Figures 9D, E for CA1 SR and CA3 SR, respectively. Ball-and-chain phagocytic microglia showed a significant correlation with plaque density in CA3 SR (Figure 9E, R^2^ = 0.37, significant) but not in CA1 SR (Figure 9D, R^2^ = 0.141, not significant). From the data reported in Figure 9E, it appears that in CA3 SR of Tg-T mice (white circles), at the same density of ball-and-chain microglia, amyloid plaques were lower than in Tg mice (black circles), confirming that the treatment boosts the protective effects of microglia in Tg-T mice. The same is not true in CA1 SR of Tg-T mice (Figure 9D). Figure 9C shows the quantitative analysis of the percentage of ball and chain microglia on plaques in CA1 and CA3 SR of Tg and Tg-T mice. In CA3 SR of Tg and Tg-T mice, a signifiucantly high percentage of ball-and-chain microglia was located around plaques in comparison to CA1 SR (** *P* < 0.01 Tg CA3 vs. Tg CA1 and Tg-T CA3 vs. Tg-T CA1), showing that microglia in CA3 is more reactive than in CA1.

In CA1 and CA3 SR of Tg and Tg-T mice, astrocytes are more numerous and more activated around plaques, as shown in the representative confocal image of Figures 9F, F1 in which many activated astrocytes (GFAP positive, green) surround an Aβ plaque (red). Astrocytes surrounding the plaque with their branches were much bigger than those far from the plaque, indicated by the open arrows. The quantitative analyses, performed in CA1 SR and CA3 SR of Tg and Tg-T mice, demonstrated that in CA3 SR of Tg-T mice, the treatment increased significantly the percent of astrocytes located around plaques (Figure 9G, Tg-T + 140% vs. Tg). Statistical analysis carried out by two-way ANOVA with Area and Treatment as the two variables revealed that there was a significant main effect for Treatment, F (1, 24) = 33.89, *P* = 0.0001, Area, F (1, 24) = 9.16, *P* < 0.027, and Interaction Treatment-Area: F (1, 24) = 18.28, *P* = 0.00358. Bonferroni post test showed that there was a significant difference between areas (*P*< 0.001, CA3 vs. CA1), and treatment with pre-and pro-biotics significantly increased astrocytes around plaques only in CA3 SR of Tg-T mice (*P* < 0.001, Tg-T vs. Tg).

Figure 9H shows a representative confocal image with triple immunohistochemistry of astrocytes (GFAP, red), microglia (IBA1, green) and Aβ (blue) taken at the boundaries between CA3 SP and SR of a Tg-T mouse. It is evident that activated astrocytes and ball-and-chain microglia surrount the Aβ plaqueand ball-and-chain microglia phagocytose Aβ (open arrow). Far from the plaques microglia and astrocytes have a phenomic typical of non-reactive cells.

## 4 Discussion

In this study, we evaluated the effects that a 6-month diet enriched in pre- and probiotics might have on neurodegeneration and on the histopathological hallmarks of AD in the hippocampus of APP/PS1 mice, a cerebral area involved in the pathogenetic mechanisms of AD. It has been demonstrated that at this age APP/PS1 mice have well developed plaques and already show the pathophysiological signs of AD (Radde et al., 2006). We performed a thorough analysis, encompassing the evaluation of plaque density, neuronal degeneration and glia phenomic modifications, and differentiated the effects in CA1 and CA3 hippocampal areas, which are known to respond differently to insults. Interestingly, we found that treatment with pre- and probiotics decreased Aβ plaque deposition, improved neuroprotection, and caused modification of astrocytes and microglia phenomic, mainly in CA3, and to a lesser extent in CA1 hippocampus.

We found that the treatment with pre- and probiotics significantly decreased the density of Aβ plaques in CA3 SR while in CA1 the reduction was not significant. Neuronal damage in CA1 SP was significantly prevented in Tg-T mice, while no damage was found in CA3. These are remarkable results, indicating that a diet additioned with pre- and probiotics might be of help in the prevention of neurodegeneration, especially if started early in life and in subclinical conditions such as mild cognitive impairment. Neurons and glia not only cooperate to maintain the physiology of the nervous system, but are also in continuous crosstalk with the microbiota and gut (MB-gut-brain axis) that produce molecules necessary for the correct functionality of the brain (Sharon et al., 2016), so much that dysbiosis contributes to several neurodegenerative disorders, including Alzheimer’s disease (AD) (Mayer and Tillisch, 2011; Sharon et al., 2016; Friedland and Chapman, 2017; Sun et al., 2020). The contribution of each bacterial strain to the integrity/dysfunction of brain mechanisms are still quite unknown. We do know that MB is the main producer of Aβ in the body (Galloway et al., 2019) and the changes of MB composition with age can explain the progressive age-dependent increase of Aβ production (Galloway et al., 2007; Galloway et al., 2008; Galloway et al., 2009; Cattaneo et al., 2017; Sun et al., 2020), suggesting the existence of a strong relationship between MB changes and AD etiopathogenesis. Many studies show that most AD patients have dysbiosis (Friedland and Chapman, 2017; Hung et al., 2022), and a recent meta-analysis (Hung et al., 2022) indicates that changes of the gut MB may occur early in the disease process before the beginning of neurodegeneration (Li et al., 2019; Liu et al., 2019; Hung et al., 2022). In accordance to these findings, probiotic supplementation rich in Gram-positive bacteria improves cognition in patients with AD (Akbari and Hendijani, 2016). Furthermore, the Gram-positive *B. subtilis* and the Gram-negative *E. coli* are the main producers of Aβ (de Kivit et al., 2014; Pistollato et al., 2016; Friedland and Chapman, 2017), and APP/PS1 transgenic mice have altered gut bacteria and overproduce Aβ (Harach et al., 2017). The ability of the intestinal cells to handle molecules of bacterial origin explains why and how these molecules have access to the brain, using bottom-to-top directional pathways, causing beneficial or pathological effects depending on their properties (Kujala et al., 2011).

In Tg mice supplemented with pre- and probiotics, astrocytes were activated in CA3 hippocampus mainly around plaques. It is well known that physiologically the MB transforms tryptophan from the diet to produce aryl hydrocarbon (AH) ligands that bind to astrocyte AH receptors (Zelante et al., 2013; Rothhammer et al., 2016), thus modulating astrocyte activity towards a protective phenotype with anti-inflammatory properties (Wikoff et al., 2009; Zelante et al., 2013). These findings suggest that microbial metabolites of dietary tryptophan can modulate the anti-inflammatory status of astrocytes and contribute to neuroprotection. Astrocyte reactivity could promote outgrowth and survival of neurons, synaptogenesis, as well as phagocytosis (Liddelow and Barres, 2017). We hypothesize that in Tg-T mice, astrocytes shifted towards a protective phenotype and cooperated with reactive microglia (Paolicelli et al., 2022) in the scavenging of Aβ plaques, especially in CA3 hippocampus as also demonstrated in a different mouse model of AD (Ugolini et al., 2018). Indeed, we found that in CA3 SR of Tg-T mice, a high proportion of reactive astrocytes were surrounding Aβ plaques and cooperated with microglia in the scavenging of Aβ. The hippocampus, primarily affected in AD, is particularly susceptible to the products of the MB such as short chain fatty acids (SCFAs) (Sharon et al., 2016) that can reach the brain through the circulation, cross the BBB, and target microglia to regulate their function. It has been shown that prebiotics (dietary fibres) can modulate the expression of bacterial proteins associated with SCFAs production (Wong et al., 2006). Furthermore, a diet with low content of fibres causes reduction of bacterial SCFAs products in mice (Sonnenburg et al., 2016) human primates (Nagpal et al., 2018) and humans (Duncan et al., 2007). Interestingly, decreased production of SCFAs (Unger et al., 2016) has been found in neurodegenerative diseases. Furthermore, immune cells expressing receptors for MB-deriving SCFAs can migrate to the brain through the BBB (Wang et al., 2018).

Our results show that microglia changed their morphological features around amyloid plaques in a region-specific way, being highly differentiated in CA3 SR to form ball-and-chain CD68+ structures, that characterize microglia diversification towards a phagocytic phenotype (Vidal-Itriago et al., 2022), and expressed CX3CR1, the fraktalkine receptor. Since it has been demonstrated that microglia have age- and region-dependent transcriptional identities (Hart et al., 2012; Grabert et al., 2016), it is possible that they have an “immune-vigilant” phenotype in CA3, explaining their higher activation in response to Aβ plaques, and to the treatment with pre- and probiotics, giving rise to a protective phenotype. In APP/PS1 mice, that have a dysbiotic MB, (Harach et al., 2017), reactive microglia migrate towards Aβ plaques, interact with Aβ and regulate Aβ levels in the brain (Holmes et al., 2003; Cunningham et al., 2005), while Germ Free APP/PS1 mice have significantly less microglia cells. Thus, it appears that signals from MB delineate microglia phenomic, and MB modifications alter microglia functionality. Furthermore, in APP/PS1 mice, modification of gut MB is related to increased brain infiltration of Th1 cells (Wang et al., 2019), while ablation of gut MB blocks brain infiltration of Th1 cells and activation of microglia, indicating that gut MB is functional in promoting Th1/microglia inflammatory mechanisms in AD pathogenesis (Wang et al., 2019). The MB, shaping the brain innate immune system, conditions the maturation and functionality of microglia (Erny et al., 2015) which, surveying brain parenchyma and maintaining tissue homeostasis (Prinz and Priller, 2014) have a role in coordinating the responses between the immune system and cognitive functions (Kettenmann et al., 2013; Schafer and Stevens, 2013; Safaiyan et al., 2016). In the APP/PS1 mice, the treatment with pre- and probiotics was able to prevent all the biological and metagenomic changes characteristic of this transgene (Traini et al., 2024), further demonstrating the efficacy of this treatment.

The protective capacity of microglia decreases considerably in a proinflammatory context (Koenigsknecht-Talboo and Landreth, 2005; Block et al., 2007). Accordingly, in the APP-SL70 mouse, a transgenic model of AD generated on a C57BL/6J genetic background that coexpresses KM670/671NL mutated amyloid precursor protein (APP) and L166P mutated presenilin (PS) one under the control of a neuron-specific Thy1 promoter, and with congophylic Aβ plaques starting from 5 to 6 months of age, phagocytic activity of microglia inversely correlates with Aβ plaque deposition and aging (Blume et al., 2018). On the contrary, our results demonstrated that the phagocytic activity of microglia, evidenced by the quantitative analysis of ball-and-chain cells, significantly correlated with the density of Aβ plaques, mainly in CA3 hippocampus. These results indicate that a healthier and more physiological microbiota, as can be found in our transgenic mice treated with pre- and probiotics (Traini et al., 2024), may send messages to the brain that modulate microglia reactivity. We hypothesize that in Tg-T mice microglia cells shifted towards a neuroprotective phenotype and cooperated with astrocytes in the scavenging of Aβ plaques, especially in CA3 hippocampus.

Recently, many experimental studies indicate that microglia, as neurons, exist physiologically as heterogeneous, mixed populations, which differ in their transcriptomic and morphofunctional characteristics. Microglia show unique morphological, ultrastructural, and physiological features in the different hippocampal regions (Tan et al., 2020; Lana et al., 2023), and between the two hippocampal poles. Microglia are anything but static cells, and they are exceptionally responsive to alterations in the surrounding environment with a vast array of receptors that form a “sensome”, which survey their surroundings and allow detection and response to different negative or positive stimuli (De Felice et al., 2022; Paolicelli et al., 2022). Microglia phenomics vary in different brain areas and in CA1 and CA3 hippocampus they respond differently to stimuli (Grabert, et al., 2016; Ugolini et al., 2018). For instance, in CA1 of aged rats, microglia are less numerous (Cerbai et al., 2012), are irregularly distributed, and have reduced cytoplasmic projections (Wong, 2013; Deleidi et al., 2015; Lana et al., 2016; Lana et al., 2019) than in CA3. The comparison between microglia phenomic modifications in CA1 and CA3 can give insights into the higher sensitivity of CA1 to neurodegenerative insults, both in experimental animal models and in humans (Mueller et al., 2010; Small et al., 2011; Bartsch and Wulff, 2015). In this respect, Vinet and coworkers demonstrated that even ramified microglia exert neuroprotective roles, and this function is region-specific, at least in the hippocampus (Vinet et al., 2012). However, the exact phenomics of microglia and their differential responses to different stimuli are still a matter of investigation.

The cluster of differentiation 68 (CD68) is recognized as a general marker of reactive phagocytic microglia (Perego et al., 2011). Early accumulation of CD68-positive microglia, mainly at Aβ deposition sites, participate in clearing Aβ and are usually associated to reduction of Aβ levels. Aβ deposition followed by a gradual phenomic modification and increase of phagocytic microglia accumulation at Aβ deposit sites was demonstrated (Matsumura et al., 2015). Indeed, in our experiments we showed that ball-and-chain microglia were highly positive for CD68 in CA1 and CA3 SR of APP/PS1 mice. In Tg-T mice, the treatment slightly increased this effect in CA3 SR, decreased it in CA1 SR, but increased it in CA1 SP. In CA3 of Tg mice, the expression of CX3CR1 was significantly higher than in Wt and Wt-T mice, and further increased in Tg-T mice. On the contrary, CX3CR1 did not change significantly in CA1 SR of any of the experimental groups. These findings, taken together, suggest that reactive, highly mobile, microglia cells (see Morsch et al., 2015) moving from SR to SP and cooperating with activated astrocytes may help clearing plaques in this area (see also Figure 9H), decreasing the neurodegenerative effect of Aβ plaques.The role of fraktalkin and its receptor CX3CR1 in health and disease are still a matter of debate and their action may differ depending upon the pathophysiological condition and spatial localization of microglia. On one hand, defective fraktalkin-CX3CR1 interactions have been associated with neuroinflammatory disorders. On the other one it has been demonstrated that depletion of CX3CR1 in mice decreases Aβ deposition in mouse models of AD, possibly caused by decreased phagocytic activity of microglia (Lee et al., 2010). On the contrary, in the adult brain the activation of CX3CR1 contributes to glutamatergic synaptic transmission and plasticity, cognition, and regulation of inflammatory responses [see (Eugenín et al., 2023)]. All these discrepancies may depend on the different models used and the different brain regions studied.

Our results once more support the hypothesis that in CA3 hippocampus, microglia express a high efficient scavenging activity, regulate Aβ load via phagocytosis and exert a more effective neuroprotection than in CA1. In accordance to the literature (Sierra et al., 2010), we demonstrated that the spherical pouches of ball-and-chain microglia phagocytosed Aβ. Furthermore, ball-and-chain phagocytic microglia showed a significant correlation with plaque density in CA3 SR, but not in CA1 SR. All these results, taken together, demonstrated that pre- and probiotics can boost the phagocytic activity of microglia and decrease the load of Aβ in the hippocampus in a region specific way, with the higher activity in CA3 SR.

Indeed, experimental studies that modulate the gut microbiota by the use of prebiotics and/or probiotics to maintain its healthy composition or reboost it, demonstrate effects on cognitive functions and behavior in different animal models. Therefore, nutraceutical/pharmacological intervention on gut microbiota is emerging as a promising strategy for managing neurodegenerative diseases.

## Scope

The gut microbiota (MB) is an elective target of intervention with prebiotics and probiotics that can maintain or recover its physiological composition. The main objective of the present research was to verify whether and how a diet enriched in prebiotics and probiotics, administered chronically starting at 2 months of age to APP/PS1 mice, a transgenic mouse model of AD, modify AD histopathological hallmarks.

This research has significant novelties: first, evaluate whether treatment with pre- and probiotics could modify Abeta plaques and neurodegeneration in hippocampal CA1 and CA3 areas of APP/PS1 mice; second, prebiotics and probiotics are devoid of side effects and maintaining or recovering gut-MB composition, could represent a nutraceutical strategy to prevent or reduce AD.

The beneficial effects of the treatment, aimed to preserve the MB, one of the MB-gut-brain axis components, confirm the strict interrelation among all of them to ameliorate the brain conditions. Because of the absence of side effects, the use of pre and probiotics represents a light approach with strong potentialities in efficacy.

## Data Availability

The raw data supporting the conclusions of this article will be made available by the authors, without undue reservation.
